# Process optimization and antioxidant activity of white peony root components extracted via ultrasound-assisted deep eutectic solvents

**DOI:** 10.1016/j.ultsonch.2025.107685

**Published:** 2025-11-17

**Authors:** Li Xin, Ammara Sohail, Zihan Li, Yan Cheng, Yilu Wang, Li Cui, Hidayat Hussain, Zheng Wang, Wenshuang Zhao, Jinhua Du, Yue Li, Jixiang He, Daijie Wang

**Affiliations:** a School of Pharmaceutical Sciences, Shandong University of Traditional Chinese Medicine, Jinan 250014, China; b Food Resources Development and Health Product Creation International Joint Laboratory/Biological Engineering Technology Innovation Center of Shandong Province, Heze Branch of Qilu University of Technology (Shandong Academy of Sciences), Heze 274000, China; c Department of Chemistry, University of Okara, Okara 56300, Pakistan; d Shandong Analysis and Test Center, Qilu University of Technology (Shandong Academy of Sciences), Jinan 250014, China; e Shandong Jinsheng Biological Technology Co. LTD, Linyi 276629, China; f Department of Genetics and Cell Biology, Basic Medical College, Qingdao University, Qingdao, Shandong 266071, China

**Keywords:** *Paeonia lactiflora* Pallas, Box-Behnken design, Antioxidant components, Reusability, Response surface method

## Abstract

The study aimed to extract antioxidant-rich components from white peony root (WPR) using the ultrasound-assisted deep eutectic solvents (UAE-DES) and optimizing them through response surface methodology to enhance their antioxidant potential. Twenty DESs and three conventional solvents were evaluated, and choline bromide-formic acid (DES17) was found best among them for extracting antioxidant components from WPR. The UAE-DES process was optimized using the Box-Behnken response surface method, achieving a maximum yield (18.16 ± 0.67 mg/g) under optimal conditions (41 min, 1:40 g/mL, and 58 % DES/H_2_O), with an extraction efficiency 4–13 times higher than traditional techniques. The study also evaluated the significant DES reusability and recovery of antioxidant components using macroporous resins. Additionally, the DES-derived optimized WPR extract demonstrated significant *in vitro* antioxidant activity. FTIR, ^1^H NMR, SEM, and DFT calculations were carried out to better understand chemical component interactions and extraction mechanisms. This study advances green solvent chemistry and ultrasound applications in natural product processing by providing a viable, environmentally friendly method for the quick and effective extraction of important phytochemicals from plant matrices.

## Graphical abstract

**Figure:**
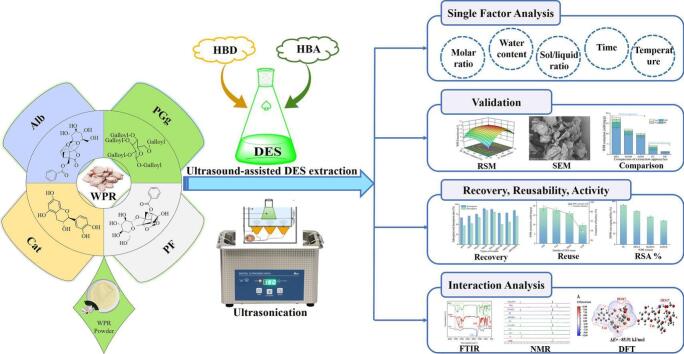

## 1 Introduction

White peony root (WPR), the dried root of *Paeonia lactiflora* Pallas, is a popular herbal drug in traditional Chinese medicine and has potential as a functional ingredient in health-promoting foods and nutraceuticals [1]. WPR is gaining interest in the food industry due to its health benefits, including antioxidant, anti-inflammatory, and immune-modulating properties [2,3]. Its rich phytochemical profile makes it a valuable natural resource for developing functional ingredients. WPR offers a sustainable and culturally acceptable source of bioactive compounds for health-oriented food products [4]. Pharmacological research has shown that the major bioactive components of WPR are monoterpene glycosides (MTGs), primarily including paeoniflorin (PF) and albiflorin (Alb), as well as polyphenolic compounds (PPs), such as catechin (Cat) and 1,2,3,4,6-penta-*O-*galloyl-β-ᴅ-glucose (PGg). These bioactive compounds offer promising prospects for the development of nutraceuticals and functional foods that promote wellness and prevent illness [5,6]. As a result, WPR bioactive components (PF, Cat, Alb, and PGg) have garnered increased attention due to their numerous potential health benefits, with the extraction of PPs and MTGs playing a crucial role. Therefore, effective and environmentally friendly extraction techniques should be used to facilitate the widespread availability of WPR components.

Despite its promise, it remains challenging to extract these chemicals from WPR efficiently. The past 20 years have seen the development of several pretreatment techniques that isolate and purify PPs and MTGs from the WPR using traditional solvents. However, traditional solvents like methanol, acetone, or ethanol are highly volatile, toxic, and non-renewable; their usage as extractants is harmful to both the environment and consumers [7]. These restrictions prevent WPR from being used in food applications on a large, sustainable scale. To fully realise WPR's potential as a functional food ingredient, it is imperative to develop extraction methods that are efficient, economical, and environmentally friendly. To improve the effectiveness of the extraction process and lessen its detrimental effects on the environment, an alternative green solvent system that uses an inventive approach is desperately needed.

The deep eutectic solvent (DES) has garnered considerable interest recently as a potential new green solvent for the extraction of PPs and MTGs due to its unique extraction efficiency [8]. Research has shown that deep eutectic solvent-assisted extraction (DAE) produces natural PPs and MTGs with greater yields and more potent biological activity than old extraction techniques [9]. To increase the extraction efficiency of natural PPs and MTGs from WPR, physical auxiliary extraction methods, such as ultrasound-assisted extraction (UAE), have also been employed in addition to the extraction solvent [10]. UAE techniques are more efficient, require less energy, and have a shorter extraction time than traditional techniques [11]. Although previous studies have explored the application of DES in the extraction of *Paeonia lactiflora*’s root components [12,13], but those works mainly concentrated on specific compounds or optimised extraction conditions for specific bioactives. In contrast, this study methodically develops and optimises a new ultrasound-assisted deep eutectic solvent (UAE-DES) technique designed especially to maximize the extraction efficiency of a variety of bioactive substances (PF, Cat, Alb, and PGg) from WPR and obtain natural antioxidants.

In this study, twenty types of DESs, both binary and ternary, were evaluated for extracting PF, Cat, Alb, and PGg from the WPR of *Paeonia lactiflora*, in comparison with conventional solvents. Response surface methodology (RSM) optimized the extraction parameters, and macroporous resin was used to recover components from the DES. Biological assessments of free radical scavenging further highlighted the potential uses and importance of the findings. Notably, the study provided a fresh perspective on specific solvent-target drug interactions by combining molecular dynamics simulations and NMR technologies for the first time, thereby clarifying the network of interactions between hydrogen bond donors (HBDs) and hydrogen bond acceptors (HBAs) in DESs and WPR targeted components. The extraction mechanism was further investigated using Fourier transform infrared spectroscopy (FT-IR) and scanning electron microscopy (SEM). This study not only provides a theoretical foundation for the optimal use of WPR extracts but also gives fresh insights into the innovation and ecological sustainability of natural product extraction methods.

## 2 Materials and methods

### 2.1 Materials and chemicals

The WPR was purchased from a nearby traditional Chinese medicine shop in Jinan, China. It was filtered via a standard 60 μm mesh screen after being ground by Yongli Scientific Instrument Co., Ltd. (Ruian, China). The reference compounds, which comprised catechins (Cat), paeoniflorin (PF), albiflorin (Alb), and 1,2,3,4,6-penta-O-galloyl*-β*-d-glucose (PGg), were purchased from Shanghai Yuanye Bio-Technology Co., Ltd. (Shanghai, China).

Sinopharm Chemical Reagent Co., Ltd. supplied the following compounds: choline chloride (98.0 %), citric acid (98.0 %), propionic acid (99.0 %), and ethylene glycol (98.0 %) used in the synthesis of DES. Lactic acid (85.0 %), glycerol (99.0 %), acetic acid (99.8 %), formic acid (96.0 %), DPPH, ABTS^+^, and FRAP reagents were purchased from Tianjin Kemiou Chemical Reagent Co., Ltd. (Tianjin, China). The following materials were purchased from Shanghai Macklin Biochemical Co., Ltd. (Shanghai, China): malonic acid (98.0 %), urea (99.0 %), glutaric acid (99.0 %), choline bromide (98.0 %), ZnCl_2_ (99.0 %), MgCl_2_ + 6H_2_O (98.0 %), glucose (99.0 %), ʟ-proline (99 %), xylitol (99.0 %), and sulphuric acid (98.0 %). We purchased macroporous resins, HPLC-grade acetonitrile, and phosphoric acid from Tedia Company Inc., US. Double-distilled water was made using a Milli-Q water purification system from Millipore, Bedford, Massachusetts, USA. The remaining substances were all preferably analytically graded.

### 2.2 HPLC conditions

**Fig. 1:**
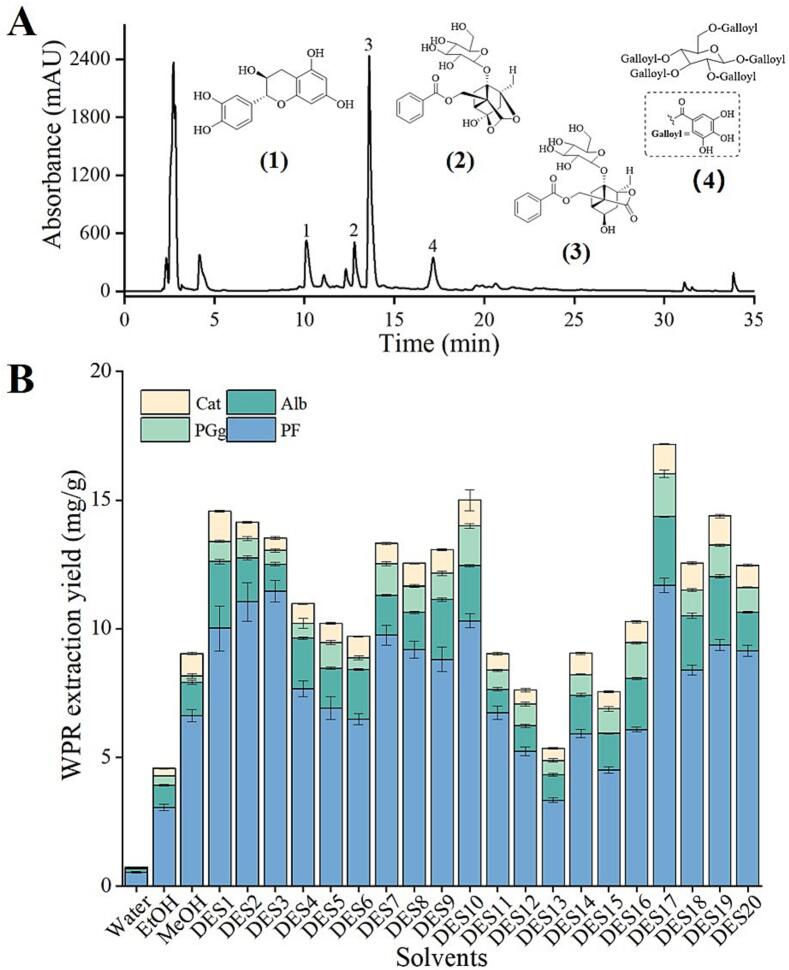
A) HPLC chromatogram of white peony root. The numbers represented the following compounds: 1. Catechin (Cat), 2. Albiflorin (Alb), 3. Paeoniflorin (PF), and 4. 1,2,3,4,6-penta-*O*-galloyl*-β*-ᴅ-glucose (PGg). B) Effect of different DESs and conventional solvents on the extraction yield of WPR via UAE. The error bars indicate the mean (±) SD (*n* = 3).

An HPLC method was established for the simultaneous determination of four major constituents, namely paeoniflorin (PF), catechin (Cat), albiflorin (Alb), and 1,2,3,4,6-penta-O-galloyl-*β*-d-glucose (PGg) in white peony roots (Fig. 1A). An Agilent series 7C HPLC system (Agilent Technologies, Waldbronn, Germany) was employed and equipped with a vacuum degasser, a quaternary pump, an autosampler, a UV detector, and a column compartment. Compounds were separated on an Eclipse XDB-C_18_ column (4.6 × 250 mm i.d., 5 μm particle, Agilent). Gradient elution of the analytes was performed using acetonitrile (A) and 0.1 % phosphoric acid-H_2_O (B). Initial conditions were 5 % A (0–5 min), 9–15 % A (5–8 min), 15–25 % A (8–23 min), 25–35 % A (23–28 min), 35–90 % A (28–35 min). The column temperature was maintained at 25 °C, and the flow rate was 1.0 mL/min. The detector wavelength was set at 230 nm.

Standard solutions of target compounds with known concentrations were initially made to begin the compound identification procedure. Chromatograms were then recorded when the samples and these standard solutions were put into an HPLC apparatus. By comparing the retention durations of the sample chromatograms with those of the reference substances, the peaks were located. HPLC software was used to determine the area under each peak. By creating calibration curves and plotting the concentration of the standard solutions against the relevant peak regions, quantitative analysis was carried out. By entering the peak area into the corresponding calibration curve, the concentration of each constituent (PF, Cat, Alb, and PGg) in the WPR was ascertained.

### 2.3 Preparation of DESs

Suitable hydrogen bond acceptors (HBAs) and hydrogen bond donors (HBDs) were taken to prepare DESs based on earlier reports [14]. The appropriate HBDs and HBAs were weighed according to a specific molar ratio, then they were added to a 50 mL flask and thoroughly mixed. Following this, the flask was placed in a magnetic stirrer set to a constant temperature of 80 °C and stirred for 1–4 h, until a clear, homogeneous liquid was formed. In the end, the reaction had been stopped, removed, and allowed to cool to ambient temperature before being preserved in a sealed dryer for later use. Supplementary Table S1 lists the chemicals that were utilized to prepare DESs with suitable molar ratios.

### 2.4 Ultrasonic-assisted extraction process

After drying and crushing, the WPR was sieved to a 60-mesh size. A 2 mL centrifuge tube containing 100 mg of defatted WPR powder was precisely weighed, 1 mL of eutectic solvent was added (1:10 g/mL Solid-liquid ratio), and the mixture was vortexed to first filter out the DES. UAE was performed by ultrasonic irradiation (KQ2200B, Kunshan Ultrasonic Instrument Co., Ltd., China) at 30 °C for 30 min, followed by centrifugation at 7500 r/min for 7 min. WPR constituents were isolated from the supernatant and filtered through a 0.22 μm organic filter. PF, Cat, Alb, and PGg in WPR were then quantified using an HPLC by using above mentioned conditions.

### 2.5 Determination of chemical constituent content in WPR

#### 2.5.1 Establishment of standard curves

To create the calibration curves, four standard compounds were taken, including PF, Cat, Alb, and PGg, because they were majorly present in WPR according to the literature [15]. Using an analytical balance (ME104, Mettler Toledo) to weigh 7 mg of each standard exactly, then dilute it with methanol in a 7 mL volumetric flask until it hits the scale line to make a 1 mg/mL standard solution (mother liquor). Standard solutions were made with concentrations of 0.05, 0.1, 0.16, 0.2, 0.4, and 1 mg/mL, in that order. For analysis under the aforementioned chromatographic conditions, 10 μL of the standard solution at various concentrations was pipetted and injected into the liquid chromatograph one after the other. Each concentration was analyzed in triplicate using the same HPLC conditions as described above. A calibration curve was constructed by plotting the peak area ratio of each compound to that of the internal standard against the concentration of each compound. After fitting the regression equation and analyzing the data, the linear equations for standard compounds Cat, Alb, PF, and PGg were found to be “*Y*_cat_ = 62791*x* – 140.25, *Y*_Alb_ = 23665*x* + 1.9592, *Y*_PF_ = 27779*x* – 192.38, and *Y*_PGg_ = 40480*x* + 0.9871″ with ”*R*_cat_^2^ = 0.9994, *R*_Alb_^2^ = 0.9981, *R*_PF_^2^ = 0.9991, and *R*_PGg_^2^ = 0.9993, respectively.

#### 2.5.2 Determination of sample content

The standard curve was used to quantify the PF, Cat, Alb, and PGg's content in WPR, and all experiments were done three times. Using the following formula, the extraction yield of PF, Cat, Alb, and PGg contents in WPR was determined:

(1) $$Y=\frac{∁\times d\times V}{m}\;\;\;\;\;\;\;\;\;\;\;$$

where *Y* represented the Total WPR extraction yield (mg/g); *C* denoted the conc. of the sample (mg/mL); *d* showed the dilution factor of the sample; *V* represented the total volume of sample liquid (mL); *m* represented the mass of the sample (g).

### 2.6 Single-factor experiments

To identify the ideal processing conditions using DESs, this experiment examined the effects of DES type, DES molar ratio, DESs/H_2_O mass ratio, ultrasonic temperature, ultrasonic duration, and solid–liquid ratio on the PF, Cat, Alb, and PGg extraction rate extracted from WPR. The UAE experiment was repeated three times and averaged the results. The experimental design for the single factor is described in Table S2.

### 2.7 Optimal design of response surface

The Box-Behnken experimental design model was used to optimize the response surface experiment based on the analysis of the previously described single-factor experimental findings. Three highly influential factors were identified, namely the solid–liquid ratio, ultrasonic time, and DESs/H_2_O content. Three factors and three levels were then used to optimize the process conditions; the factors and levels are displayed in Table S3. The total extraction yields of WPR contents as the response values *Y* in RSM were statistically compared using GraphPad Prism 5.01 for Windows (GraphPad Software, San Diego, CA, USA).

### 2.8 Recycling of WPR constituents and reuse of DES

The static adsorption and desorption capabilities of WPR components for AB-8, MQ-1, HPD-100, D101, MQ-08, MQ-10, HPD-750, and HPD-BJQH macroporous resins were assessed using the extracted solution. The conical flasks (25 mL) were filled with 12 mL of water, 4 g of pretreated resin, and 0.1 g of WPR sample. Equation (2) was used to calculate the rate of adsorption after the flasks were placed on a constant temperature oscillator (NHWY-200B, Changzhou Nuoji Instrument Co., Ltd) for 24 h at 30 °C to allow for adsorption. Methanol was added for desorption when the resins were transferred to new conical flasks, and Equation (3) was used to calculate the rate of desorption. After a careful analysis of the adsorption and desorption rates, the macroporous resin with the best adsorption and separation performance was identified. The most effective macroporous resin discovered throughout the screening process was further evaluated using dynamic adsorption and desorption tests for the recovery of WPR components from DES. By initially eluting the DES with water and then a gradient concentration of 70 % ethanol, the bioactive components of WPR were recovered. The quantities of WPR components in the recovered solutions were determined using the process described in Section 2.2. The WPR components were then extracted using the DES once again to assess its effectiveness and reusability across several cycles.

(2) $$\mathrm{Adsorption}\left(\%\right)=\frac{C_{o}V_{o}-C_{1}V_{1}}{C_{o}V_{o}}\times 100$$

(3) $$\mathrm{Desorption}\left(\%\right)=\frac{C_{2}V_{2}}{C_{o}V_{o}-C_{1}V_{1}}\times 100$$

where *C_1_* and *V*_1_ indicated the concentration (mg/mL) and volume (mL) of the sample solution following adsorption, whereas *C_2_* indicated the concentration (mg/mL) and *V_2_* indicated the volume (mL) of the sample solution following desorption. *C_o_* and *V_o_* stood for the concentration (mg/mL) and total volume (mL) of the sample solution before adsorption.

### 2.9 Determination of antioxidant activity

This study assessed the antioxidant activity of WPR extracts of various solvents to investigate WPR extracts as natural antioxidant sources that can be utilised as substitutes for synthetic antioxidants in food supplements or nutraceuticals. Using previously published protocols [16], the antioxidant capacity of the WPR extracts was evaluated using three complementary tests: 2,2-diphenyl-1-picrylhydrazyl (DPPH) radical scavenging activity, ferric reducing antioxidant power (FRAP), and 2,2′-azinobis (3-ethylbenzothiazoline-6-sulfonic acid) (ABTS) radical cation decolorization.

#### 2.9.1 DPPH assay

To prepare a 20 μg/mL DPPH solution, 2.0 mg of DPPH was dissolved in ethanol and diluted to 100 mL. Sample solutions with concentrations of 0.5, 1, 2, and 4 mg/mL were prepared. The experiment utilized three groups: the sample group (3 mL DPPH solution + 2 mL sample solution), the control group (3 mL ethanol + 2 mL sample solution), and the blank group (3 mL DPPH solution + 2 mL ethanol). All groups were kept in the dark for 30 min, and then absorbance was measured at 517 nm. Ascorbic acid (Vitamin C) served as the positive control at concentrations of 2, 4, 6, and 8 μg/mL. Radical scavenging activity and IC_50_ values were calculated from the absorbance measurements. The following formula was used to calculate the DPPH radical scavenging ability (RSA) of WPR extracts:

$$DPPHRSA\left(\%\right)=\frac{A_{0}-\left(A_{i}-A_{j}\right)}{A_{0}}\times 100\%$$

where A_0_ indicated the absorbance of the blank group; A_i_ represented the absorbance of the sample group; A_j_ indicated the absorbance of the control group.

#### 2.9.2 ABTS assay

An ABTS radical cation solution (7 mM ABTS, 2.45 mM potassium persulfate in 50 % ethanol + water) was prepared and incubated overnight at 25℃. The solution's absorption was adjusted to 0.700 ± 0.005 at 734 nm using 50 % ethanol. The experimental design included a sample group (6 mL ABTS solution + 1 mL sample), a control group (6 mL ABTS + 1 mL water), and a blank group (6 mL water). Absorbance at 734 nm was measured to assess radical scavenging activity and IC_50_ values. The following formula was used to calculate the ABTS radical scavenging ability (RSA) of WPR extracts:

$$ABTSRSA\left(\%\right)=\frac{A_{0}-\left(A_{i}-A_{j}\right)}{A_{0}}\times 100\%$$

where A_0_ indicated the absorbance of blank group; A_i_ represented the absorbance of sample group; A_j_ indicated the absorbance of control group.

#### 2.9.3 FRAP assay

To each sample solution (20 µL), 180 µL of FRAP reagent was added, which was prepared by mixing 10 mmol/L tripyridyltriazine solution, 0.3 mol/L sodium acetate buffer (pH 3.6), and 20 mmol/L FeCl_3_·6H_2_O solution in a volume ratio of 1:10:1, followed by incubation in a 35 °C water bath for 30 min. The reaction mixture was then incubated in the dark at 30 °C for 10 min. The absorbance (*y*) was measured at a wavelength of 593 nm. The ferric reducing antioxidant power (FRAP) was expressed as mmol FeSO_4_ concentration (*x*), based on the FeSO_4_ standard curve: *y* = 0.7691*x* + 0.0121, *R*^2^ = 0.9995.

### 2.10 Comparison of different extraction methods

#### 2.10.1 Ultrasound-assisted extraction (UAE)

Using a slightly modified version of the previous UAE method, the UAE extracted the PF, Cat, Alb, and PGg from WPR using conventional solvents such as 70 % ethanol and methanol. The mixture was centrifuged for 7 min at 7500 r/min after extraction, and the precipitate was discarded to yield the extract. Using the method described in Section 2.2, the extraction rates of the WPR components were determined after the extract was filtered through a 0.45 μm microporous membrane.

#### 2.10.2 Maceration extraction (MC)

After being weighed, one gram of dry WPR powder was added to a 25 mL conical flask. Following a 36 h maceration at 25 °C with 10 mL of 70 % ethanol, the material was centrifuged for 7 min at 7500 r/min. Once the precipitate was disposed of, the resulting extract was filtered through a 0.45 μm microporous membrane [17]. The extraction rates of WPR components were then determined using the method described in Section 2.2.

#### 2.10.3 Percolation extraction (PC)

4 g of carefully weighed WPR powder was placed into a glass percolation cylinder. After soaking in 70 % ethanol for 15 h, the sample was allowed to percolate at a rate of 3 mL/min while being maintained at 25 °C [18]. Following the collection of the filtrate and its passage over a 0.45 μm microporous membrane, the process described in Section 2.2 was used to determine the extraction rates of the WPR components

### 2.11 Experimental methods for the extraction mechanism

Using a Supra^TM^ 55 SEM (Zeiss, Germany), morphological characterization of the WPR following ultrasonic-assisted extraction was carried out. The samples were deposited on aluminum stubs and sputtered with a thin layer of gold before being scanned with an in-lens SE detector. The ultrahigh tension was set at 5.00 kV, and the magnification was 500× [11]. Using KBr pellets for solid samples like choline bromide and smears for liquid samples such as DES ChBr-FA and formic acid, the FTIR spectra of the WPR extracts were obtained using an FTIR spectrophotometer. The scanning procedure involved 32 scans in the 400–4000 cm^−1^ range with a spectral resolution of 4 cm^−1^ [19]. Using nuclear magnetic resonance hydrogen spectroscopy (Bruker, Rheinstetten, Germany) with tetramethylsilane (TMS) as the internal standard and DMSO‑*d_6_* as the solvent, the presence of hydrogen bonds in the ideal eutectic solvent was further characterized. The chemical shift (δ) value was expressed in *ppm*.

### 2.12 DFT analysis

Density functional theory (DFT) simulations were performed using the Gaussian 16 software package to evaluate the molecular interactions between deep eutectic solvents (DES) and WPR components. The B3LYP hybrid exchange–correlation functional with the 6-31G(d) basis set was used for geometry optimization and frequency computations. The lack of imaginary frequencies validated the global minima of all optimised geometries. The Multiwfn 3.6 software was used for wavefunction analysis and electrostatic potential fitting, while VMD 1.9.3 was used to visualise electrostatic potential (ESP) maps [20]. The following Equation (4) can be used to calculate the interaction energy between different molecular segments.

(4) $$\Delta E_{\mathrm{DES}-(Cat/Alb/PF/PGg)}=E_{\mathrm{DES}-\left(\mathrm{Cat}/Alb/PF/PGg\right)}-\left(E_{\mathrm{DES}+}E_{\mathrm{Cat}/Alb/PF/PGg}\right)$$

Here, *E*_DES-(Cat/Alb/PF/PGg)_ represented the energy of a DES and WPR components, whereas *E*_DES_ and *E*_Cat/Alb/PF/PGg_ were the energies of the isolated molecules DES (ChBr-FA) and WPR components.

### 2.13 Data processing

Data processing and analysis were performed using Origin Pro 2021 and Design-Expert 13 software. To assess statistical significance (*p* < 0.05), one-way analysis of variance (one-way ANOVA) and the Student's *t*-test were used. Each test was conducted three times (*n* = 3).

## 3 Results and discussion

### 3.1 Screening of eutectic solvent types

The determination of the extraction yield for the targeted compounds was performed through HPLC analysis using a previously established method [21]. Previous studies have indicated the presence of various constituents in the WPR, including flavonoids, polyphenols, and monoterpenes glycosides [22]. In this study, four compounds (Cat, Alb, PF, and PGg) were extracted from WPR using DESs as extraction solvents. Fig. S1 displays the calibration curves for these standard compounds.

Twenty eutectic solvents, including both binary (two-component) and ternary (three-component) systems, were synthesized for the ultrasonic extraction of PF, Cat, Alb, and PGg from WPR. As demonstrated by Fig. 1B, eutectic solvents, particularly the mix of choline bromide and formic acid (DES17, a binary solvent), were more effective in extracting PF, Cat, Alb, and PGg than conventional solvents. Chemical components with higher polarity are simpler to extract using ChBr-FA (1:2) by the concept of similarity and solubility [23]. DES17 (ChBr-FA, 17.17 ± 0.482 mg/g) was the most extractable of the DESs that were evaluated; it outperformed methanol in terms of extraction efficiency and was followed by DES10 (ChCl-Gly, 15.00 ± 0.834 mg/g), DES1 (ChCl-Aa, 14.57 ± 1.051 mg/g), and DES19 (ChBr-EG-FA, 14.38 ± 0.377 mg/g). By comparison, the lowest extraction efficiency was shown by DES5 (ChCl-Xyl, 10.20 ± 0.59 mg/g) and DES15 (ChCl-Glu-Mg, 7.56 ± 0.276 mg/g). Interestingly, water (0.73 ± 0.047 mg/g) barely extracted PF, Cat, Alb, and PGg from WPR. By comparison of three types of HBA (Choline bromide, choline chloride, and ʟ-proline) with formic acid (HBD), the best DES for PF, Cat, Alb, and PGg extraction was binary ChBr-based DES, which was somewhat nontoxic and reasonably priced (Supplementary Table S1). Therefore, DES17 was chosen to be the extraction solvent in the subsequent studies.

### 3.2 Analysis of single-factor experiments

#### 3.2.1 Effect of molar ratio on WPR component extraction rate

**Fig. 2:**
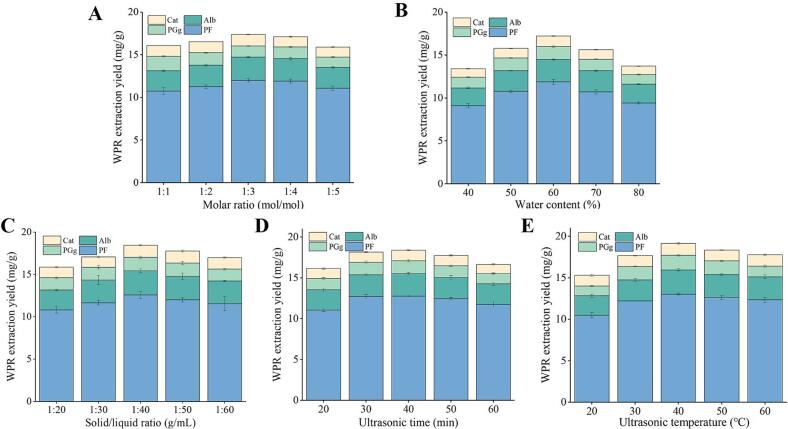
Effects of different A) molar ratio of DES17 (ChBr-FA), B) water content (%), C) solid/liquid ratio (mg/mL), D) ultrasonic time (min), and E) temperature (^o^C) on the extraction yield of WPR components via UAE. The error bars indicate the mean (±) SD (*n* = 3).

Fig. 2A illustrates how the molar ratio of the HBD and HBA affects the WPR components extraction yield. WPR total extraction rate peaked at a molar ratio of 1:3 mol/mol between choline bromide and formic acid and then declined as the molar ratio of formic acid increased. As a result, the ideal molar ratio for the HBD (formic acid) and HBA (choline bromide) was 1:3 mol/mol, and this molar ratio was used for further optimization.

#### 3.2.2 Effect of DES/water content on WPR component extraction rate

DESs often exhibit high viscosity and low diffusion capacity, which hinders their ability to permeate into the sample matrix and reduces the extraction rate of the desired components [24]. Although adding water to the eutectic solvent might lower its viscosity, the ideal DESs/H_2_O mass ratio also varies depending on the particular component. Fig. 2B illustrates the impact of DESs/H_2_O content on the WPR extraction rate. The findings demonstrated that when the DESs/H_2_O content increased from 40 % to 60 %, WPR components extraction had the largest rise. The rate of WPR extraction decreased when the DESs/H_2_O content rose over 60 %. It's possible that the first injection of water caused the eutectic solvent's viscosity to drastically drop and its diffusion ability to rise, which in turn increased the extraction rate. The high-water content of the eutectic solvent disrupted the hydrogen bonds between its constituents when the mass ratio of DESs/H_2_O reached 60 % to 80 %. This decreased the contact force between the DES and WPR contents, and decreased the amount of target components [25]. For the following studies, a DES with H_2_O content of 60 % was used for further experiments.

#### 3.2.3 Effect of solid**/**liquid ratio on WPR components extraction rate

Fig. 2C illustrates how the solid–liquid ratio affects the WPR components extraction yield. The greatest rate of WPR extraction occurred at a 1:40 g/mL solid–liquid ratio; as this ratio increased from 1:40 g/mL to 1:60 g/mL, the rate of extraction declined noticeably. Reducing the amount of WPR powder sample in the eutectic solvent will also decrease the extraction efficiency [26]. Thus, 1:40 g/mL was the ideal solid–liquid ratio for this experiment.

#### 3.2.4 Effect of ultrasonic time on WPR components extraction rate

Another important factor in the extraction of natural compounds is extraction time. Fig. 2D illustrates how quickly WPR constituents are extracted using optimum ultrasonic time. The rate at which WPR components were extracted increased between 20 and 40 min, peaked at that point, and then gradually declined as the time frame was extended from 40 min. WPR constituents’ degradation may be the cause of the decreasing extraction yield. Therefore, it was found that 40 min was the appropriate extraction time.

#### 3.2.5 Effect of ultrasonic temperature on WPR components extraction rate

Temperature during extraction has an impact on mass transfer and, consequently, chemical composition [10]. As seen in Fig. 2E, the total WPR extraction yield increased as the extraction temperature rose from 20 °C to 40 °C and then declined as the extraction temperature climbed further. One possible explanation is that as the temperature of the extraction rises, the DES's viscosity falls, accelerating molecular movement, strengthening mass transfer, and increasing the diffusion rate [27]. All of these factors increase the rate at which WPR constituents were extracted, and when the temperature of the extraction rose above 40 °C, the heat-sensitive WPR constituents broke down, lowering the extraction rate. Consequently, the ideal extraction temperature was considered to be 40 °C.

### 3.3 Analysis of the experimental results of RSM

#### 3.3.1 Establishment of response model and significance analysis

Based on the previously reported single-factor experiments, it was shown that three variables significantly affected the effectiveness of PF, Cat, Alb, and PGg isolated from WPR: the DESs/H_2_O content%, the solid/liquid ratio, and the ultrasonic duration. The components and levels of the response surface design are displayed in Table S3. The Box-Behnken (central combination) of the response surface of Design-Expert 13.0.1.0 software (Stat-Ease, USA) was utilized to create a three-factor, three-level experiment to determine the ideal extraction process parameters. To determine the experimental error, three sets of repeated zero-point tests and five sets of center-point trials were employed. Table S4 displays the Box-Behnken experimental design scheme and response surface findings, whereas Table 1 displays the ANOVA of the regression model derived from the response surface experimental data.

**Table 1:** ANOVA of regression models for WPR extraction rate using the ChBr-FA.

Source of variance	Sum of squares	degree of freedom	Mean square deviation	*F*-value	*p*-value*
Model	33.69	9	3.74	145.32	<0.0001*
*A*	0.4186	1	0.4186	16.25	0.0049*
*B*	0.4802	1	0.4802	18.64	0.0035*
*C*	0.0105	1	0.0105	0.4081	0.5433
*AB*	1.66	1	1.66	64.61	<0.0001*
*AC*	0.002	1	0.002	0.0786	0.7873
*BC*	0.1521	1	0.1521	5.9	0.0454*
*A^2^*	20.9	1	20.9	811.26	<0.0001*
*B^2^*	3.19	1	3.19	123.8	<0.0001*
*C^2^*	4.32	1	4.32	167.66	<0.0001*
Residual	0.1803	7	0.0258		
Lack of Fit	0.0706	3	0.0235	0.8586	0.5312
Pure Error	0.1097	4	0.0274		
Sum	33.87	16			
*R^2^*	0.9947				
*R^2^_Adj_*	0.9878				
*C.V.%*	0.9893				

*Significant if *p* < 0.05.

Using Design-Expert 13 software, the regression analysis was carried out on the data obtained in the table, and the quadratic multinomial regression equation with the total extraction yield (*Y*) of WPR components as the objective function was obtained by regression fitting of each factor:

$$\begin{array}{c} Y=18.16+0.2287A-0.2450B-0.0362C-0.6450AB\\ +0.0225AC+0.1950BC-2.23A^{2}-0.8703B^{2}-1.01C^{2} \end{array}$$

**Fig. 3:**
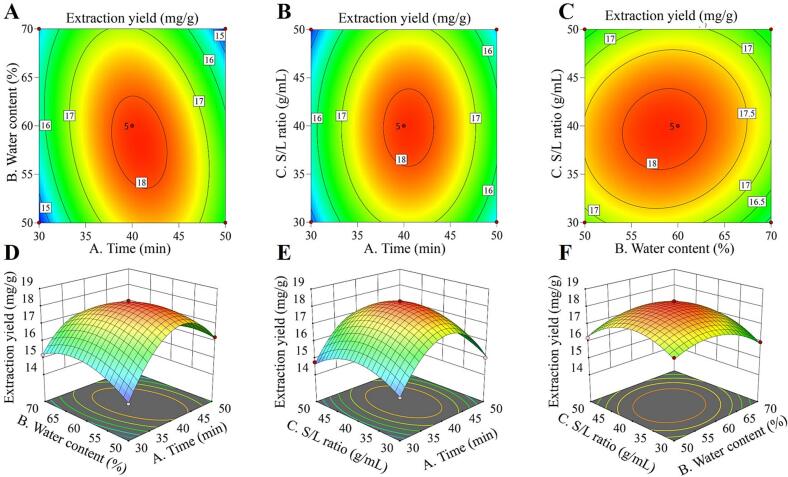
A-C) 2D contour plots and D-F) 3D response surface graphs of the interaction of various factors on the extraction rate of WPR components.

The ANOVA of the experimental results in Table 1 showed that the model fits successfully and effectively, as evidenced by the *F*-value of 145.32 for the model and 0.8586 for the misfit term. Additionally, the model's *p-*value was < 0.0001, indicating that the quadratic multinomial regression model had high significance and could effectively analyze the relationship between independent variables and objective functions. At the same time, it can be seen from Table 1 that *A* (ultrasonic time) and *B* (water content) had a significant effect on *Y* (total WPR extraction yield) (*p* < 0.05), while *C* (solid–liquid ratio) had no significant effect on *Y* (*p* > 0.05). The *p* < 0.05 of the *AB* and *BC* interaction terms indicated that their interaction had a significant effect on the extraction yield of PF, Cat, Alb, and PGg. The effects of *A^2^, B^2^*, and *C^2^* on the response value reached a highly significant level (*p* < 0.05). Model plots, such as 3D response surface plots and contour plots, are used to show how each independent variable affects the response value (Fig. 3). Furthermore, the model's correlation coefficient (*R^2^* = 0.9947) and adjustment coefficient (*R^2^_Adj_* = 0.9878) showed that the data was trustworthy and the model fitted the data well. With a coefficient of variation (*C.V.*%) of 0.9893, the model demonstrated strong accuracy, high credibility, and good repeatability. As a result, the model may be utilized to optimize the extraction of PF, Cat, Alb, and PGg from WPR. From the *F*-value, it can be seen that the influence of each factor on the extraction rate of WPR is in the order of water content > ultrasonic time > solid–liquid ratio.

#### 3.3.2 Establishment of optimal process conditions and model validation

The solid/liquid ratio of 1:39.66 g/mL, the ultrasonic duration of 40.76 min, the DESs/H_2_O content of 58.27 %, and the estimated WPR extraction yield of 18.19 mg/g were the ideal extraction conditions determined by RSM. Validation experiments (*n* = 3) were conducted under these circumstances to confirm the accuracy of the experimental design and the efficacy of the regression model. Three parallel extraction tests were conducted under real conditions (*A* = 41 min; *B* = 58 %; *C* = 1:40 g/mL), and the average value was computed to demonstrate that the total WPR extraction yield was 18.16 ± 0.67 mg/g. The study's optimal process parameters are dependable and confirm the validity of the experimental design since the relative error between the actual (18.16 ± 0.67 mg/g) and anticipated value (18.19 mg/g) was minimal.

### 3.4 Recovery of WPR constituents and DES

**Fig. 4:**
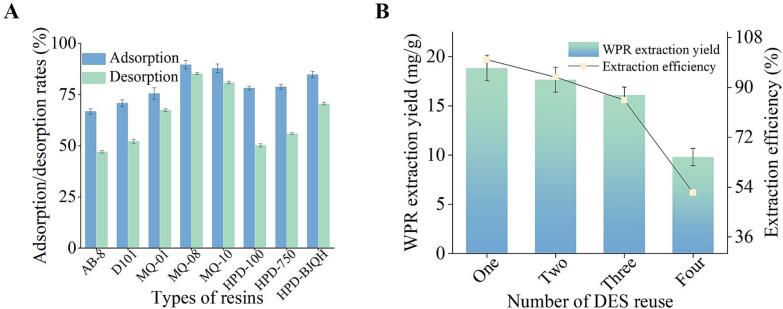
A) WPR adsorption/desorption rates on various resins. B) DES reusability. Data are represented as mean ± SD (*n* = 3).

Because of DES's high water miscibility and low vapor pressure, recovering target chemicals from DES-extracted solutions is difficult. Macroporous resins are one of the most often used recovery techniques [28]. Eight different types of macroporous resins were tested for adsorption and desorption efficiencies: D101, AB-8, MQ-1, MQ-08, MQ-10, HPD-100, HPD-750, and HPD-BJQH. The results are displayed in Fig. 4A. Both the adsorption and desorption rates were maximum for MQ-08 macroporous resin (89.62 ± 1.98 % and 85.23 ± 0.48 %, respectively). These findings led to the selection of MQ-08 for dynamic adsorption and desorption tests, which ultimately resulted in a recovered WPR component content of 76.38 %. Fig. 4B illustrates the reuse of DES. In the first three uses, the total extraction yield stayed over 80 % of the original rate; however, in the fourth use, there was a notable decrease, suggesting that DES might be used at least three times.

### 3.5 Active component content and in vitro antioxidant activity measurement

**Fig. 5:**
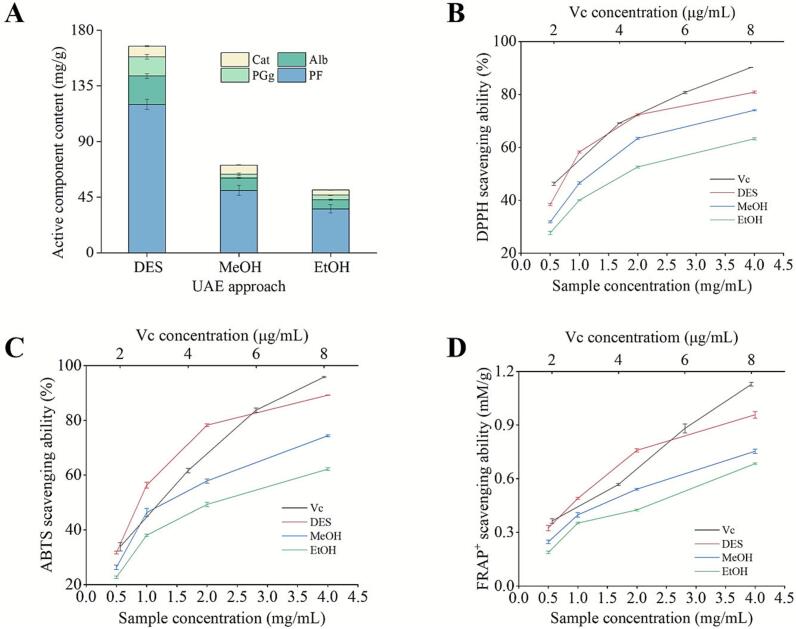
A) The impact of UAE treatment using various solvents on the content of WPR active components. B-D) Antioxidant activity of WPR extracts obtained with different solvents (DES, Methanol, and Ethanol), assessed by DPPH, ABTS^+^, and FRAP assays. Data are represented as mean ± SD (*n* = 3).

To determine the concentration of extracted active components from WPR extracts, a comparison test between a low-melting-point solvent (DES, ChBr-FA) and traditional organic solvents (methanol/ethanol) was carried out under similar ultrasonic conditions. Significantly, the amounts of bioactive components in WPR extracts were impacted by the different solvents. In comparison to methanol and ethanol ultrasonication, the concentration of active components in the WPR extracts during DES ultrasonication was noticeably greater (Fig. 5A).

Furthermore, the antioxidant capacity of several WPR extracts was assessed in this investigation using three antioxidant indices (DPPH, FRAP, and ABTS). The findings showed that the WPR extract treated with DES ultrasonication exhibited considerably higher DPPH, FRAP, and ABTS^+^ radical scavenging activity compared to those produced with conventional solvents under similar optimized ultrasonication settings (Fig. 5B-D). Notably, the antioxidant activity of the DES extract was significantly greater than that of methanol and ethanol extracts across all assays, with IC_50_ value of DPPH (0.80 ± 0.01 mg/mL *vs* 1.21 ± 0.05 mg/mL *vs* 1.56 ± 0.01 mg/mL), and IC_50_ of ABTS^+^ (0.80 ± 0.02 mg/mL *vs* 1.10 ± 0.06 mg/mL *vs* 1.83 *vs* 0.04 mg/mL). Despite this improvement, these extracts demonstrated much lower activity than Vc, with IC_50_ of DPPH (3.44 ± 0.18 μg/mL *vs* 0.80 ± 0.01 mg/mL), and IC_50_ of ABTS^+^ (4.23 ± 0.44 μg/mL *vs* 0.80 ± 0.02 mg/mL) (Table 2). However, these findings suggest that DES enhances extraction efficiency, extracting the highest WPR active components content and producing higher antioxidant-potential extracts compared to conventional solvents, demonstrating enhanced free radical neutralization and iron-reducing capabilities in the extracted WPR components. This may be explained by DES's capacity to enhance active ingredient solubility, while ultrasonication's cavitation impact helps break down plant cell walls, which increases the release of bioactive components from WPR [13]. In conclusion, the WPR DES ultrasonication extract showed the highest antioxidant capacity, highlighting its potential for applications in nutraceuticals, health benefits, and the development of food chemistry research.

**Table 2:** Antioxidant capacity of WPR extracts.

Extracts	Antioxidant capacity	
	DPPH IC_50_	ABTS IC_50_
Vc	3.44 ± 0.18 μg/mL	4.23 ± 0.44 μg/mL
DES	0.80 ± 0.01 mg/mL	0.80 ± 0.02 mg/mL
MeOH	1.21 ± 0.05 mg/mL	1.10 ± 0.06 mg/mL
EtOH	1.56 ± 0.01 mg/mL	1.83 ± 0.14 mg/mL

### 3.6 Comparison of modern *vs* traditional extraction techniques

**Fig. 6:**
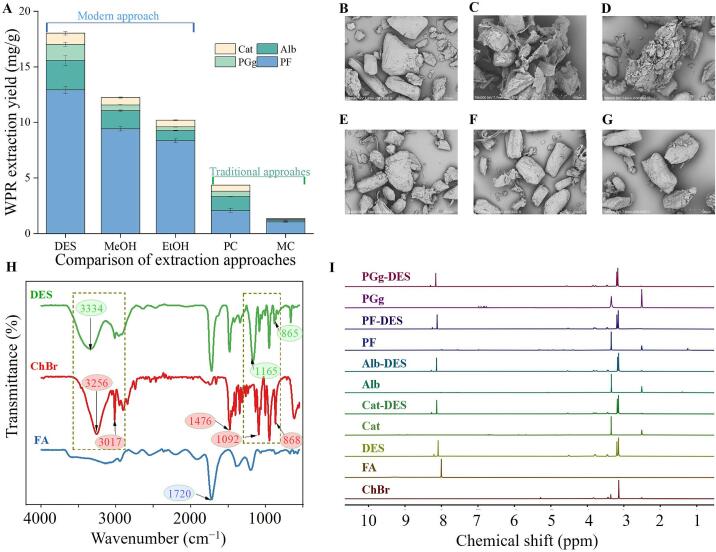
A) Comparison of extraction approaches for WPR. SEM analysis of raw WPR powder B) Untreated, C) ultrasonic-assisted eutectic solvent extraction, D) ultrasonic-assisted methanol extraction, E) ultrasonic-assisted ethanol extraction with 1:40 g/mL S/L for 40 min at 40°C, F) percolation extraction (PC), and G) maceration extraction (MC). H) FTIR spectra of choline bromide (ChBr), formic acid (FA), and DES (ChBr-FA). I) ^1^H NMR spectra of choline bromide (ChBr), formic acid (FA), DES (ChBr-FA), catechins (Cat), Albiflorin (Alb), Paeoniflorin (PF), 1,2,3,4,6-penta-*O*-galloy-*β*-ᴅ-glucose (PGg), and mixed with eutectic solvents.

To demonstrate the superiority of the UAE-DES technique, the effects of several methods, such as UAE-DES, UAE-MeOH, UAE-EtOH, PC, and MC, on the extraction yield of WPR components were compared. The results are shown in Fig. 6A. The outcome unequivocally demonstrates that the UAE-DES method has the highest WPR extraction yield, which is approximately 1.4–1.7 times higher than UAE-MeOH (1.7 times of Alb, 1.5 times of Cat, 1.3 times of PF, and 2.9 times of PGg) and UAE-EtOH (3.0 times of Alb, 1.6 times of Cat, 1.5 times of PF, and 4.9 times of PGg). Similarly, UAE-DES method also significantly outperforms traditional extraction techniques, with yields approximately 4–13 times higher for PC (2.1 times of Alb, 1.8 times of Cat, 6.2 times of PF, and 3.0 times of PGg) and MC (18.7 times of Alb, 12.0 times of Cat, 12.1 times of PF, and 25.6 times of PGg). The results revealed that DES and UAE have shown improved extraction efficiency compared to traditional solvents and conventional methods. This may be attributed to the fact that DES efficiently breaks down cellulose, allowing for effective cellulose dissolution. The interaction between HBA (ChBr) and HBD (FA) with cellulose chains enhances the dissolution process, resulting in higher extraction yields of PF, Cat, Alb, and PGg. Likewise, the UAE also outperforms conventional techniques due to its cavitation effect, enabling quick extractions and improved repeatability. These findings are consistent with previous reports [13].

### 3.7 Structural characterization

#### 3.7.1 SEM analysis

SEM imaging was used to analyze the appearance of raw WPR samples extracted using various solvents (DES17 (choline bromide-formic acid), ethanol, and methanol) by different extraction approaches (UAE, PC, and MC) to evaluate the extraction mechanism. Before extraction, the WPR raw sample powder was undamaged, its textural structure was evident, and there were no indications of damage (Fig. 6B). The surface of WPR was severely degraded following the application of ultrasound-assisted DES therapy, resulting in full rupture and visible porosity (Fig. 6C). Following UAE with traditional solvents like methanol and ethanol, the tissue surface of WPR somehow developed some cracks and holes, and the sample particles became rough (Fig. 6D & E). Nevertheless, the sample surface was somewhat damaged during both PC and MC extraction (Fig. 6F & G). Among all of them, UAE-DES caused more destruction to the WPR sample matrix. These findings demonstrated that DES, with their ease of disruption, are effective in destroying cell walls, releasing substance components, and can enhance extraction efficiency when coupled with ultrasonication [29].

#### 3.7.2 FTIR spectroscopy

The FTIR analysis of choline bromide, formic acid, and ChBr-FA revealed that the formation of eutectic solvents relies on hydrogen bond formation. We acquired FT-IR spectra of choline bromide, formic acid, and ChBr-FA to verify the interactions between ChBr-FA, as shown in Fig. 6H. The characteristic absorption bands of ChBr at 3256.14 cm^−1^ and 3017.37 cm^−1^ corresponded to O-H and C-H telescopic vibrations, while the strong absorption band of FA at 1720.87 cm^−1^ was attributed to the stretching vibration of C=O, confirming the properties of choline bromide and formic acid, respectively [30,31]. The IR spectra of the prepared eutectic solvent (DES17) overlapped with choline bromide and formic acid, with broadening and shifting peaks in the DES system, especially the corresponding amino vibration band (865.96–1165.94 cm^−1^). The O-H stretching vibration in DES indicated hydrogen bond formation, while the C-H stretching vibration observed at 3017.37 cm^−1^ disappeared, confirming hydrogen bonding. Moreover, the main peaks of formic acid and choline bromide could be seen in the DES IR spectra, which showed that the functional groups of the reactants stayed stable during the process.

#### 3.7.3 ^1^H NMR spectroscopy

^1^H NMR studies were performed on different systems to further investigate the interaction between the HBA (ChBr) and the HBD (FA) in DES and the target compounds (Cat, Alb, PF, and PGg). The hydrogen bond formation decreases electron shielding on active hydrogen atoms, leading to a decrease in electron cloud density and a shift in resonance absorption towards a higher chemical shift [32]. Fig. 6I illustrates the analysis of ChBr, FA, DES, Alb, Cat, PGg, PF, and solutions of DES with target compounds (Alb, Cat, PGg, PF). The results revealed that the resonance signal at 4.92 ppm in the DES spectrum indicated the formation of an H-bond between the ammonium ion of ChBr and the hydroxyl group of FA, increasing the chemical shift of the active hydrogen signal and shifting it to the downfield. At the same time, all the peaks in the DES system could be assigned to their individual components, and no signal of side reactions was observed, indicating that DES was formed by non-covalent interaction (see Supplementary Table S5). Furthermore, ^1^H NMR was used to examine the interaction between the target compounds and the chosen DES (ChBr-FA). The active hydroxy-proton signals of targeted compounds (PF, Cat, Alb, and PGg) moved to the downfield region upon addition to the DES, indicating hydrogen bond formation. Furthermore, the proton signals from the glycosidic and phenolic hydroxyls of target compounds became substantially weaker or vanished in the DES solutions of their DES spectra, probably due to active proton exchange, permit H-D exchange. The study found that significant interactions between DES and target molecules enhanced ChBr-FA's extraction performance, indicating that target antioxidant compounds were successfully extracted from the produced DES.

#### 3.7.4 DFT analysis

Based on the previous FTIR results, it can be concluded that no formation of new chemical bonds or changes in functional groups occurred during the DES extraction process. It can thus be inferred that the observed phenomena are attributed solely to weak interactions. Based on this fact, it is predicted that the target compound may associate with DES molecules via weak intermolecular forces.

**Fig. 7:**
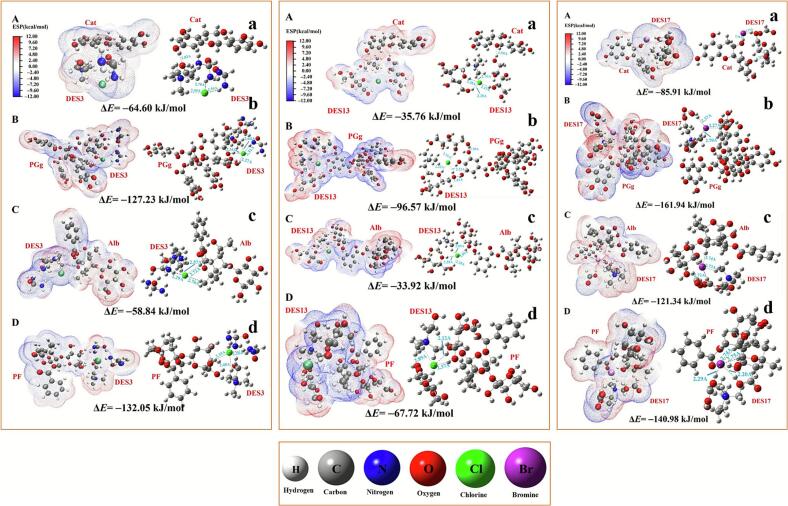
Molecular interaction between DES and WPR components. A-D) Electrostatic potential surface map; a-d) detailed interaction and bond lengths between DESs (DES3.5.17) and WPR components (Cat, PGg, Alb, and PF). (DES 3: Choline chloride-Urea; DES5: Choline chloride-Xylitol; DES17: Choline bromide-Formic acid).

From another perspective, the analysis of FTIR spectra confirmed the involvement of X–H groups in the system in the formation of hydrogen bonds, though it could not provide precise geometric structural information. In fact, based on the series of potential hydrogen bonding sites present, multiple initial guess structures were designed and iteratively optimized using Gaussian 16. After screening, DES3, DES5, and DES17 were retained as reasonable initial models. In the experiments, all the designed candidate structures were ultimately observed to fluctuate near the binding site depicted in Fig. 7. It can therefore be concluded that, within this explicitly bounded system, the structure illustrated in the figure occupies a statistically thermodynamically favoured position. Moreover, the fitted electrostatic potential (ESP) clearly indicated the directionality of hydrogen bonding, demonstrating that hydrogen bonds tend to form along the direction of the negative ESP gradient. This outcome provides dual verification for the rationality of the hydrogen bonding configuration in our model.

Geometries of all species were fully optimized without symmetry constrains. The electrostatic potential surface (ESP) maps (Picture A.B.C.D.) displayed varied charge distributions, with the red region representing positive ESP and the blue area representing negative ESP. The molecular interaction diagram (Picture a.b.c.d) illustrates the number and strength of hydrogen bonds generated by DES3.5.17 with the target chemicals (Table S6). As shown in Fig. 7, DES17 exhibited the highest number and strength of hydrogen bonds with the target compound that underwent the most significant energy reduction, while DES5 showed the lowest number and weakest hydrogen bonds. This observation aligned with the experimental screening results for optimal DES performance: DES17, which induced the greatest energy decrease and formed the most and strongest hydrogen bonds, also demonstrated the highest extraction efficiency. In contrast, DES5, which presented the smallest energy reduction and the fewest/weakest hydrogen bonds, yielded the lowest extraction efficiency. Taking Cat as an example (Fig. 7Aa), the binding of different DES systems (DES3, 5, and 17) to Cat led to changes in system energy. DES17 formed two hydrogen bonds (1.76–2.36 Å) with Cat, resulting in the largest energy reduction (–85.91 kJ/mol). DES3 formed a single hydrogen bond (1.83 Å) with Cat, leading to a moderate energy decrease (–64.60 kJ/mol). DES5 also formed one hydrogen bond (1.99 Å) with Cat, but exhibited the smallest energy reduction (–35.76 kJ/mol).

The purpose of the calculations is to validate the thermodynamic stability of the association structures formed between DES and the target compounds. Based on the infrared experimental results, it can be concluded that within our model, the decrease in system energy is largely attributable to the contribution of hydrogen bonding interactions. Through a threefold assessment of hydrogen bond count, hydrogen bond strength, and system energy reduction, we can verify the differences in extraction efficacy among various DES. DES types exhibiting higher extraction rates form systems with target compounds that possess superior thermodynamic stability. This provides a sound thermodynamic rationale for our experimental observations and aligns well with our experimental data. Under otherwise identical conditions, a greater number and stronger intensity of hydrogen bonds formed between the DES and target compound result in a greater overall decrease in energy and, consequently, higher extraction efficiency.

## 4 Conclusion

The study aimed to enhance the sustainable use of WPR in health-related industries by generating high-quality bioactive extracts. For this purpose, this study used an ultrasonic-assisted eutectic solvent to extract bioactive components (PF, Cat, Alb, and PGg) from WPR, and the HPLC method was used to quantify the WPR contents. The response surface method's Box-Behnken experimental design model was used to identify three highly influential factors: DESs/H_2_O content, the ratio of solid/liquid, and the ultrasonic time. These three factors and three levels were then used to optimize the process conditions, and the ideal extraction process conditions for WPR constituents were as follows: DESs/H_2_O content 58 %, the ratio of solid/liquid of 1:40 g/mL, and ultrasonic time of 41 min. WPR extraction yield, according to the validation experiment, was 18.16 ± 0.67 mg/g. With recoveries of 76.38 %, bioactive components were effectively extracted from WPR using MQ-08 macroporous resin, and the DES could be used again for a minimum of three cycles. Comparative studies showed that UAE-DES worked better than traditional extraction techniques, increasing extraction efficiency by 4 to 13 times. DES has demonstrated significant advantages over conventional solvents in terms of enhancing the extraction yield of WPR active components and improving the radical antioxidant potential of DPPH, ABTS+, and FRAP. Moreover, spectroscopic (FTIR, ^1^H NMR, and SEM) and computational analysis (DFT) provided details on the molecular interactions and extraction mechanisms, thereby offering a more profound understanding of the procedure. Strong hydrogen bonding interactions between solute components and DES molecules protect solutes from oxidative degradation, potentially aiding in DES extraction-recovery of easily oxidizable antioxidant compounds from natural matrices. In conclusion, this work proposes an environmentally friendly and effective UAE-DES-based approach for extracting and enriching WPR components, providing insights into its mechanisms. These bioactive components hold high promise for nutritional and dietary uses, warranting further *in vivo* validation and commercial-scale testing.

## CRediT authorship contribution statement

**Li Xin:** Writing – review & editing, Writing – original draft, Formal analysis, Data curation. **Ammara Sohail:** Writing – review & editing, Writing – original draft, Formal analysis, Data curation. **Zihan Li:** Formal analysis, Data curation. **Yan Cheng:** Data curation. **Yilu Wang:** Investigation, Formal analysis. **Li Cui:** Investigation, Formal analysis. **Hidayat Hussain:** Validation, Formal analysis. **Zheng Wang:** Validation, Formal analysis. **Wenshuang Zhao:** Resources. **Jinhua Du:** Resources. **Yue Li:** Resources. **Jixiang He:** Validation, Supervision, Resources, Formal analysis. **Daijie Wang:** Writing – review & editing, Supervision, Funding acquisition, Formal analysis, Conceptualization.

## Funding

This research did not receive any specific grant from funding agencies in the public, commercial, or not-for-profit sectors.

## Declaration of competing interest

The authors declare that they have no known competing financial interests or personal relationships that could have appeared to influence the work reported in this paper.

## Data Availability

All the data are available within the article and the supplementary material.
