# Supplementary material for: Process optimization and antioxidant activity of white peony root components extracted via ultrasound-assisted deep eutectic solvents
Source: Ultrason Sonochem. 2025 Nov 17;123:107685. doi: 10.1016/j.ultsonch.2025.107685 (PMC12702406; doi:10.1016/j.ultsonch.2025.107685)
Supplement: Supplementary Data 1 [file mmc1.docx]

**Supplementary Material**

**Process Optimization and Antioxidant Activity of White Peony Root Components Extracted via Ultrasound-Assisted Deep Eutectic Solvents**

Li Xin ^1^, Ammara Sohail ^2,3^, Zihan Li ^1^, Yan Cheng ^4^, Yilu Wang ^5^, Li Cui ^4^, Hidayat Hussain ^2^, Zheng Wang ^6^, Wenshuang Zhao ^2^, Jinhua Du ^2^, Yue Li ^2^, Jixiang He ^1,^ *, Daijie Wang ^1, 2,^ *.

^1^ School of Pharmaceutical Sciences, Shandong University of Traditional Chinese Medicine, Jinan 250014, China

^2^ Food Resources Development and Health Product Creation International Joint Laboratory/Biological Engineering Technology Innovation Center of Shandong Province, Heze Branch of Qilu University of Technology (Shandong Academy of Sciences), Heze 274000, China

^3^ Department of Chemistry, University of Okara, Okara 56300, Pakistan

^4^ Shandong Analysis and Test Center, Qilu University of Technology (Shandong Academy of Sciences), Jinan 250014, China

^5^ Shandong Jinsheng Biological Technology Co. LTD, Linyi 276629, China

^6^ Department of Genetics and Cell Biology, Basic Medical College, Qingdao University, Qingdao, Shandong 266071, China

***Corresponding authors.**

*E-mail addresses*: [wangdaijie@qlu.edu.cn/wangdaijie@126.com](mailto:wangdaijie@qlu.edu.cn/wangdaijie@126.com) (D. Wang), [15628808056@163.com](mailto:15628808056@163.com) (J. He).

**Supplementary Tables**

**Table S1.** List of twenty kinds of DES and three traditional solvents for extracting WPR components after ultrasonication at 30 ^◦^C for 30 min.

| **Abbreviation** | **Comp.1** | **Comp.2** | | **Comp.3** | **Molar Ratio** | **WPR Extraction yield (mg/g)** | | | | |
| --- | --- | --- | --- | --- | --- | --- | --- | --- | --- | --- |
|  |  |  |  |  |  | **Cat** | **Alb** | **PF** | **PGg** | **Total** |
| DES1 | Choline chloride (ChCl) | Acetic acid (Aa) | |  | 1:2 | 1.17 ± 0.041 | 2.59 ± 0.098 | 10.01 ± 0.873 | 0.79 ± 0.039 | 14.57 ± 1.051 |
| DES2 | ChCl | Formic acid (FA) | |  | 1:2 | 0.64 ± 0.039 | 1.71 ± 0.074 | 11.04 ± 0.749 | 0.74 ± 0.084 | 14.14 ± 0.946 |
| DES3 | ChCl | Urea (U) | |  | 1:2 | 0.49 ± 0.059 | 1.05 ± 0.064 | 11.46 ± 0.424 | 0.53 ± 0.073 | 13.53 ± 0.62 |
| DES4 | ChCl | Propanoic acid (Pa) | |  | 1:2 | 0.77 ± 0.026 | 1.97 ± 0.047 | 7.66 ± 0.298 | 0.57 ± 0.194 | 10.98 ± 0.565 |
| DES5 | ChCl | Xylitol (Xyl) | |  | 1:2 | 0.75 ± 0.035 | 1.55 ± 0.042 | 6.91 ± 0.439 | 0.99 ± 0.074 | 10.20 ± 0.59 |
| DES6 | ChCl | Glutaric acid (Ga) | | Urea (U) | 1:1:1 | 0.82 ± 0.028 | 1.92 ± 0.043 | 6.49 ± 0.218 | 0.47 ± 0.083 | 9.70 ± 0.372 |
| DES7 | ChCl | Citric acid (Ca) | | H_2_O | 1:1:2 | 0.79 ± 0.038 | 1.54 ± 0.032 | 9.75 ± 0.381 | 1.24 ± 0.074 | 13.32 ± 0.525 |
| DES8 | ChCl | Lactic acid (La) | | Acetic acid (Aa) | 1:2:1 | 0.89 ± 0.032 | 1.44 ± 0.054 | 9.19 ± 0.329 | 1.02 ± 0.059 | 12.54 ± 0.474 |
| DES9 | ChCl | Ethylene glycol (EG) | | Formic acid (FA) | 1:2:1 | 0.91 ± 0.027 | 2.32 ± 0.069 | 8.81 ± 0.481 | 1.02 ± 0.094 | 13.07 ± 0.671 |
| DES10 | ChCl | Glycerol (Gly) | | H_2_O | 1:2:1 | 1.00 ± 0.41 | 2.14 ± 0.048 | 10.31 ± 0.283 | 1.54 ± 0.093 | 15.00 ± 0.834 |
| DES11 | ChCl | MgCl_2_.6 H_2_O (Mg) | |  | 1:1 | 0.65 ± 0.053 | 0.92 ± 0.073 | 6.73 ± 0.264 | 0.73 ± 0.043 | 9.03 ± 0.433 |
| DES12 | ChCl | Glycerol (Gly) | | ZnCl_2_ | 1:2:0.06 | 0.55 ± 0.069 | 0.99 ± 0.063 | 5.24 ± 0.172 | 0.85 ± 0.074 | 7.62 ± 0.378 |
| DES13 | ChCl | Malonic acid (Ma) | | Formic acid (FA) | 1:1:1 | 0.47 ± 0.034 | 0.99 ± 0.053 | 3.33 ± 0.089 | 0.56 ± 0.063 | 5.34 ± 0.239 |
| DES14 | ChCl | Glucose (Glu) | | H_2_O | 1:2:3 | 0.83 ± 0.045 | 1.50 ± 0.074 | 5.93 ± 0.152 | 0.79 ± 0.022 | 9.05 ± 0.293 |
| DES15 | ChCl | Glycerol (Gly) | | MgCl_2_.6 H_2_O (Mg) | 1:2:0.06 | 0.68 ± 0.028 | 1.42 ± 0.028 | 4.52 ± 0.128 | 0.95 ± 0.092 | 7.56 ± 0.276 |
| DES16 | Choline bromide (ChBr) | Urea (U) | |  | 1:2 | 0.82 ± 0.045 | 1.98 ± 0.045 | 6.09 ± 0.098 | 1.39 ± 0.048 | 10.27 ± 0.236 |
| DES17 | ChBr | Formic acid (FA) | |  | 1:2 | 1.14 ± 0.023 | 2.68 ± 0.029 | 11.68 ± 0.281 | 1.67 ± 0.149 | 17.17 ± 0.482 |
| DES18 | ChBr | Lactic acid (La) | | Urea (U) | 1:2:1 | 1.04 ± 0.064 | 2.12 ± 0.098 | 8.38 ± 0.195 | 1.00 ± 0.064 | 12.55 ± 0.421 |
| DES19 | ChBr | Ethylene glycol (EG) | | Formic acid (FA) | 1:2:1 | 1.13 ± 0.062 | 2.67 ± 0.063 | 9.37 ± 0.207 | 1.21 ± 0.045 | 14.38 ± 0.377 |
| DES20 | ʟ-Proline (Pro) | Formic acid (FA) | |  | 1:4 | 0.87 ± 0.052 | 1.50 ± 0.028 | 9.14 ± 0.218 | 0.97 ± 0.021 | 12.47 ± 0.319 |
| Traditional Solvents |  | | Water | | | 0.04 ± 0.002 | 0.13 ± 0.011 | 0.54 ± 0.032 | 0.02 ± 0.002 | 0.73 ±0.047 |
|  |  | | Ethanol | | | 0.31 ± 0.021 | 0.86 ± 0.031 | 3.05 ± 0.129 | 0.36 ± 0.012 | 4.58 ± 0.193 |
|  |  | | Methanol | | | 0.88 ± 0.052 | 1.29 ± 0.084 | 6.62 ± 0.239 | 0.25 ± 0.093 | 9.04 ± 0.468 |

**Table S2.** Single-factor experiment design.

| Number | Molar ratio | Water content | Solid/liquid ratio | Ultrasonic time | Ultrasonic temperature |
| --- | --- | --- | --- | --- | --- |
|  | mol/mol | % | g/mL | min | °C |
|  | 1:1, 1:2, 1:3, 1:4, 1:5 | 30 | 1:10 | 30 | 30 |
|  | 1:3 | 40, 50, 60, 70, 80, | 1:10 | 30 | 30 |
|  | 1:3 | 60 | 1:20, 1:30, 1:40, 1:50, 1:60 | 30 | 30 |
|  | 1:3 | 60 | 1:40 | 20, 30, 40, 50, 60 | 30 |
|  | 1:3 | 60 | 1:40 | 40 | 20, 30, 40, 50, 60 |

**Table S3.** Levels and variables of response surface analysis.

| level | factors | | |
| --- | --- | --- | --- |
|  | Time (*A*)/min | Moisture content (*B*)/% | Solid-Liquid ratio (*C*)/g·mL^-1^ |
| *–*1 | 30 | 50 | 1:30 |
| 0 | 40 | 60 | 1:40 |
| 1 | 50 | 70 | 1:50 |

**Table S4.** Scheme and outcomes of response surface analysis for WPR extraction yield

| Run | Factors | | | Response |
| --- | --- | --- | --- | --- |
|  | *A* | *B* | *C* |  |
|  | Ultrasonic time | Water content | Solid/liquid ratio | WPR Total Extraction yield  *Y* |
|  | (min) | (%, *w/w*) | (g/mL) | mg/g |
| 1 | 0 | 0 | 0 | 18.12 |
| 2 | 1 | *–*1 | 0 | 16.23 |
| 3 | 0 | 1 | *–*1 | 15.95 |
| 4 | 0 | 0 | 0 | 17.91 |
| 5 | 0 | 0 | 0 | 18.3 |
| 6 | *–*1 | *–*1 | 0 | 14.38 |
| 7 | 1 | 0 | 1 | 15.16 |
| 8 | 1 | 0 | *–*1 | 15.03 |
| 9 | 0 | 1 | 1 | 16.11 |
| 10 | *–*1 | 0 | *–*1 | 14.72 |
| 11 | 0 | 0 | 0 | 18.14 |
| 12 | 0 | *–*1 | *–*1 | 16.83 |
| 13 | *–*1 | 0 | 1 | 14.76 |
| 14 | 1 | 1 | 0 | 14.45 |
| 15 | *–*1 | 1 | 0 | 15.18 |
| 16 | 0 | *–*1 | 1 | 16.21 |
| 17 | 0 | 0 | 0 | 18.32 |

**Table S5.** ^1^H NMR spectral data of white peony root components and DES.

| Compounds | 1H-NMR data |
| --- | --- |
| Choline bromide (ChBr) | ^1^H NMR (400 MHz, DMSO-*d_6_*) δ 5.28 (t, *J* = 5.0 Hz, 0H), 3.83 (tq, *J* = 5.1, 2.5 Hz, 1H), 3.47 – 3.40 (m, 1H), 3.36 (s, 1H). |
| Formic acid (FA) | ^1^H NMR (400 MHz, DMSO-*d_6_*): δ 9.25 (s, 2H), 8.00 (s, 1H). |
| DES (ChBr-FA) | ^1^H NMR (400 MHz, DMSO-*d_6_*) δ 8.09 (s, 2H), 4.54 – 4.47 (m, 1H), 3.79 (dtd, *J* = 9.6, 6.0, 3.5 Hz, 2H), 3.49 – 3.42 (m, 1H), 3.19 (s, 4H). |
| Catechin (Cat) | ^1^H NMR (400 MHz,DMSO-*d_6_*) δ 9.18 (s, 2H), 8.94 (s, 2H), 8.87 (s, 2H), 8.82 (s, 2H), 6.75 – 6.65 (m, 4H), 6.59 (dd, *J* = 8.1, 2.0 Hz, 2H), 5.89 (d, *J* = 2.3 Hz, 2H), 5.69 (d, *J* = 2.3 Hz, 2H), 4.87 (d, *J* = 5.1 Hz, 2H), 4.48 (d, *J* = 7.5 Hz, 2H), 3.81 (tt, *J* = 7.8, 5.3 Hz, 2H), 2.66 (dd, *J* = 16.0, 5.3 Hz, 2H), 2.35 (dd, *J* = 16.1, 8.0 Hz, 2H). |
| Cat-DES | ^1^H NMR (400 MHz, DMSO-*d_6_*) δ 8.13 (s, 2H), 4.54 (dq, *J* = 4.9, 2.5 Hz, 1H), 3.86 – 3.73 (m, 2H), 3.48 – 3.41 (m, 1H), 3.18 (s, 4H). |
| Albiflorin (Alb) | ^1^H NMR (400 MHz, DMSO-*d_6_*) δ 8.06 – 7.99 (m, 2H), 7.73 – 7.63 (m, 1H), 7.55 (t, *J* = 7.7 Hz, 2H), 5.11 – 5.02 (m, 2H), 4.99 – 4.90 (m, 2H), 4.64 (d, *J* = 12.1 Hz, 1H), 4.56 (d, *J* = 12.1 Hz, 1H), 4.47 – 4.38 (m, 2H), 4.12 (s, 1H), 3.65 (dd, *J* = 10.6, 5.7 Hz, 1H), 3.39 (dd, *J* = 11.7, 5.9 Hz, 1H), 3.03 (ddt, *J* = 16.9, 13.5, 8.3 Hz, 4H), 2.78 (t, *J* = 6.3 Hz, 1H), 2.34 – 2.25 (m, 1H), 2.10 – 1.81 (m, 2H), 1.40 (s, 3H). |
| Alb-DES | ^1^H NMR (400 MHz, DMSO-*d_6_*) δ 8.14 (s, 2H), 5.26 (s, 1H), 4.54 (tt, *J* = 5.0, 2.3 Hz, 1H), 3.82 (dq, *J* = 5.4, 2.6 Hz, 1H), 3.78 – 3.71 (m, 1H), 3.47 – 3.40 (m, 1H), 3.16 (d, *J* = 11.5 Hz, 8H). |
| Paeoniflorin (PF) | ^1^H NMR (400 MHz, DMSO-*d_6_*) δ 7.99 (ddd, *J* = 7.0, 4.0, 1.4 Hz, 2H), 7.68 (tdt, *J* = 6.2, 4.8, 1.4 Hz, 1H), 7.55 (qd, *J* = 6.4, 1.7 Hz, 2H), 6.98 (s, 1H), 5.33 (s, 1H), 5.08 (d, *J* = 4.9 Hz, 1H), 5.03 – 4.88 (m, 2H), 4.71 – 4.55 (m, 2H), 4.50 – 4.35 (m, 2H), 3.65 (ddd, *J* = 11.7, 5.8, 2.0 Hz, 1H), 3.20 – 2.93 (m, 3H), 2.45 (dd, *J* = 6.6, 1.6 Hz, 1H), 2.38 (dd, *J* = 10.7, 6.8 Hz, 1H), 2.06 (dd, *J* = 11.7, 6.7 Hz, 1H), 1.82 (d, *J* = 10.6 Hz, 1H), 1.65 (dd, *J* = 12.5, 1.8 Hz, 1H), 1.29 (s, 1H), 1.25 (s, 3H). |
| PF-DES | ^1^H NMR (400 MHz, DMSO-*d_6_*) δ 8.25 (s, 1H), 8.12 (s, 3H), 4.56 – 4.49 (m, 1H), 3.79 (ddq, *J* = 12.9, 4.8, 2.5 Hz, 3H), 3.49 – 3.42 (m, 1H), 3.19 (s, 5H). |
| 1,2,3,4,6-penta-*O*-galloy-*β*-ᴅ-glucose (PGg) | ^1^H NMR (400 MHz, DMSO-*d_6_*) δ 9.24 (s, 19H), 6.97 (s, 2H), 6.91 (s, 2H), 6.83 (d, *J* = 12.7 Hz, 4H), 6.77 (s, 2H), 6.38 (d, *J* = 8.3 Hz, 1H), 5.96 (t, *J* = 9.7 Hz, 1H), 5.43 (q, *J* = 9.7 Hz, 2H), 4.59 (d, *J* = 10.3 Hz, 1H), 4.30 (s, 2H). |
| PGg-DES | ^1^H NMR (400 MHz, DMSO-*d_6_*) δ 8.30 (s, 0H), 8.16 (s, 3H), 5.29 (s, 1H), 4.56 (tt, *J* = 4.9, 2.3 Hz, 1H), 3.85 (tt, *J* = 5.2, 2.7 Hz, 2H), 3.80 – 3.74 (m, 1H), 3.49 – 3.42 (m, 1H), 3.19 (s, 5H). |

**Table S6.** Hydrogen bonding parameters of DESs-WPR components

| Entry | Sample | H-bond | H-bond distance/Å | ∠H-bond/° |
| --- | --- | --- | --- | --- |
| 1 | DES3+Cat | C10–H12···Cl22 | 2.4376 | 156.9697 |
| 2 |  | N29–H30···Cl22 | 2.7022 | 149.0161 |
| 3 |  | N38–H40···Cl22 | 2.5448 | 151.1965 |
| 4 |  | O51–H52···O8 | 1.8255 | 157.9367 |
| 5 | DES3+PGg | C14–H17···Cl22 | 2.6388 | 151.5044 |
| 6 |  | N35–H36···Cl22 | 2.3143 | 165.5417 |
| 7 |  | O115–H116···Cl22 | 2.2728 | 149.5842 |
| 8 |  | O129–H130···Cl22 | 2.3460 | 146.5410 |
| 9 | DES3+Alb | C10–H12···Cl22 | 2.5715 | 154.9504 |
| 10 |  | N35–H36···Cl22 | 2.2918 | 167.6474 |
| 11 |  | C55–H57···Cl22 | 2.7242 | 172.5087 |
| 12 |  | C100–H101···Cl22 | 2.7592 | 161.2556 |
| 13 | DES3+PF | C10–H12···Cl22 | 2.4824 | 157.8437 |
| 14 |  | N35–H36···Cl22 | 2.2041 | 174.2162 |
| 15 |  | O98–H99···Cl22 | 2.3334 | 156.6470 |
| 16 | DES13+Cat | C10–H12···Cl22 | 2.7612 | 133.2501 |
| 17 |  | O35–H36···Cl22 | 2.2838 | 157.8972 |
| 18 |  | O39–H40···Cl22 | 2.2818 | 155.8023 |
| 19 |  | O57–H58···O79 | 1.9853 | 158.7683 |
| 20 |  | O65–H66···Cl22 | 2.1326 | 164.1567 |
| 21 | DES13+PGg | O35–H36···Cl22 | 2.2195 | 162.7553 |
| 22 |  | O39–H40···Cl22 | 2.2222 | 159.0656 |
| 23 |  | O59–H60···O91 | 1.9026 | 164.5562 |
| 24 |  | O65–H66···Cl22 | 2.3153 | 163.6586 |
| 25 |  | O95–H96···O35 | 1.8099 | 152.5713 |
| 26 | DES13+Alb | C10–H12···Cl22 | 2.8312 | 133.9202 |
| 27 |  | O35–H36···Cl22 | 2.2840 | 159.4436 |
| 28 |  | O39–H40···Cl22 | 2.2750 | 154.2388 |
| 29 |  | O65–H66···Cl22 | 2.0899 | 160.6052 |
| 30 | DES13+PF | C10–H12···Cl22 | 2.8930 | 139.4224 |
| 31 |  | O35–H36···Cl22 | 2.3698 | 162.4895 |
| 32 |  | O39–H40···Cl22 | 2.1183 | 162.5908 |
| 33 | DES17+Cat | O31–H32···Br22 | 2.2534 | 151.7878 |
| 34 |  | O53–H54···Br22 | 2.3604 | 164.4435 |
| 35 |  | O64–H65···O8 | 1.7563 | 173.5278 |
| 36 | DES17+PGg | C14–H17···Br22 | 2.7007 | 158.7258 |
| 37 |  | O31–H32···Br22 | 2.3730 | 148.3108 |
| 38 |  | O93–H94···Br22 | 2.2664 | 176.6215 |
| 39 | DES17+Alb | C14–H17···Br22 | 2.7279 | 157.8984 |
| 40 |  | C28–H29···Br22 | 2.7305 | 169.3034 |
| 41 |  | C55–H56···Br22 | 2.7355 | 145.7227 |
| 42 | DES17+PF | O26–H27···Br22 | 2.1971 | 161.7086 |
| 43 |  | O31–H32···Br22 | 2.2863 | 154.1086 |
| 44 |  | C52–H53···Br22 | 2.7508 | 157.2645 |
| 45 |  | C92–H93···Br22 | 2.7421 | 143.0907 |

Both strong (O–H···Cl, O–H···Br, O–H···O) and weak (C–H···O) hydrogen bonds were listed.

**Supplementary Figures**

**
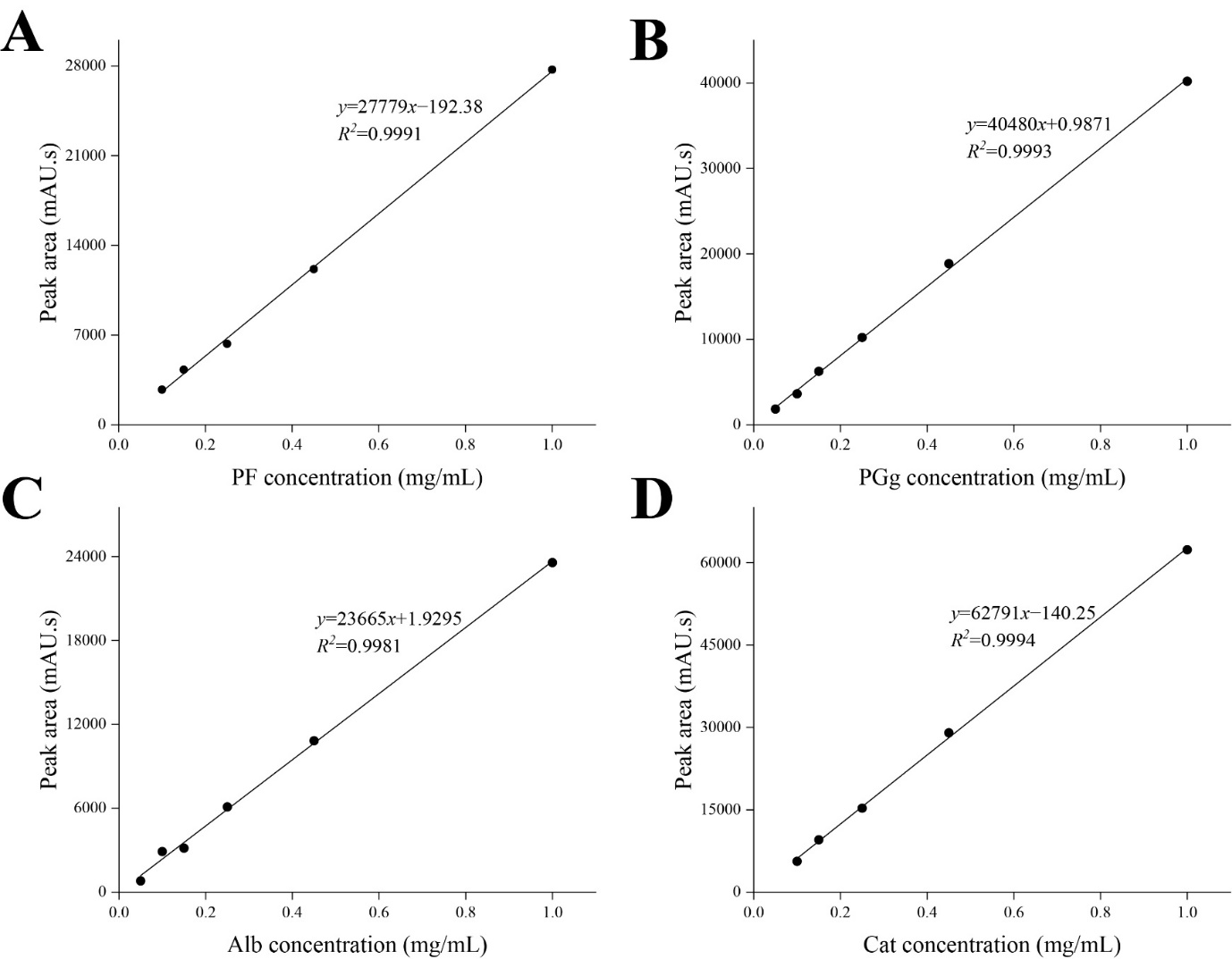
**

**Figure S1***.*  Calibration curves of standard compounds with a concentration range of 0.05–1 mg/mL: A) paeoniflorin (PF), B) 1,2,3,4,6-penta-O-galloyl-*β*-d-glucose (PGg), C) albiflorin (Alb), and D) catechin (Cat);
